# Levels of trace elements and potential toxic elements in bovine livers: A trend analysis from 2007 to 2018

**DOI:** 10.1371/journal.pone.0214584

**Published:** 2019-04-09

**Authors:** Guillaume Counotte, Menno Holzhauer, Sanne Carp-van Dijken, Jan Muskens, Deon Van der Merwe

**Affiliations:** Animal Health Services, AA Deventer, The Netherlands; Chinese Academy of Sciences, CHINA

## Abstract

Trace elements and potential toxic elements were analyzed in bovine livers submitted for autopsy in the Netherlands during the years 2007 to 2018. The age of each animal was recorded. In total, 1544 livers were analyzed for cadmium, cobalt, chromium, copper, iron, molybdenum, nickel, lead, selenium and zinc. Less than 2% of the liver samples were from veal calves. Young animals had significantly higher concentrations of iron and zinc in their livers compared to animals older than one year, while older animals had significantly higher levels of cadmium and molybdenum in their livers. Animals aged 1 to 2 years had the lowest copper and selenium levels. There was a tendency for lower chromium and nickel levels during the last years of the testing period, while copper showed an increase. Lead intoxication was only seen in the youngest group of cattle, while copper intoxication, defined as a liver copper of more than 1000 mg/kg dry matter, occurred in older animals, mainly in animals of 3 to 4 years old. This trend analysis of trace elements in bovine livers of cattle over time in recent years, and the relation of liver element concentrations with age of the animal, provides insight in the uptake and storage of these elements by cattle in The Netherlands. Possible reasons for observed trends and age-related patterns are discussed.

## Introduction

Trace elements are essential for proper functioning of humans and animals, including cattle, and inadequate intake of these elements are deleterious to health and production potential [1]. Several elements, including heavy metals and metalloids, are always present in livers and are often essential to health, even though they are toxic at high concentrations. Dysfunction of several organ systems and even mortality due to incorrect amounts of trace elements and / or toxic elements may occur [2]. Yearly, more than 3000 bovines of all ages are sent to Animal Health Services in Deventer from all regions of The Netherlands for pathological examination. When no clear cause of death is found or when the clinical history or macroscopic lesion indicate a deficiency or excess of elements, the liver is analyzed for trace elements and heavy metals.

Liver is a major storage organ for trace elements and heavy metals, and element concentrations in the liver therefore reflect exposure levels [1]. Element concentrations in the liver are interpreted as deficient, adequate, high or potentially toxic based on the age category of the animal. This is important because animals of different age classes can have different interpretation concentration ranges. Reliable reference and clinical decision values for each age category are therefore essential to the interpretation of element concentrations and the establishment of correct diagnoses.

Intake of minerals, and therefore storage of elements in liver tissue, can change over time because of changing feeding strategies and changes in the amounts and types of minerals used in feeding products. In addition, the uptake of elements from the environment (for example by ingestion of soil and grass) can change due to variation in weather, management practices and land use.

The aim of this study was to investigate trends in liver concentrations of important trace elements and potentially toxic elements in bovine livers over time and age of the animal, that may be relevant to risks of deficiencies and toxicosis.

## Materials and methods

A total of 1544 bovine livers from all regions of The Netherlands were examined post mortem during the years 2007–2018. Carcasses were sent to Animal Health Services by veterinarians to investigate the cause of death. During post mortem examination, about 500 gram of liver tissue was collected, homogenized and immediately frozen at −20°C. The livers were tested for cadmium (Cd), cobalt (Co), chromium (Cr), copper (Cu), iron (Fe), molybdenum (Mo), nickel (Ni), lead (Pb), selenium (Se) and zinc (Zn). The age of each animal was registered, and the animals were classified into six age categories. The number of animals tested per age and per year is summarized in Table 1.

**Table 1:** Number of livers analyzed for each group categorized per age and year.

year	age						
	0–7 days	1–4 weeks	2–6 month	7–12 month	13–24 month	> 2 year	total
2007	1	5	7	1	4	17	35
2008	0	3	8	1	5	29	46
2009	4	5	9	7	14	19	58
2010	16	2	9	10	9	45	91
2011	9	10	17	35	11	38	120
2012	16	9	8	21	13	53	120
2013	19	20	23	35	22	65	184
2014	9	10	12	10	13	64	118
2015	9	14	17	15	11	59	125
2016	14	20	21	19	11	91	176
2017	15	49	9	37	14	90	214
2018	31	47	29	5	13	132	257
total	143	194	220	132	153	702	1544

Element concentrations were determined with Inductively Coupled Plasma Mass Spectrometry (ICP-MS). The method was briefly as follows: after thawing, at least 50 grams of liver was homogenized. Dry matter (dm) of the sample was determined by drying for 4 hours at 103°C. The dry matter content of the livers was, on average, 0.271 gram per gram (Standard error = 0.032 gram per gram). From each liver, a duplicate sample (about 1 gram of material per sample) was digested with 6 mL 65% nitric acid using a microwave oven (temperature 200°C, maximum pressure 40 MPa, 4 min ramp time, 5 min hold time, medium stirring) (Discover SP microwave Synthesizer, CEM Microwave Technology Dublin Ireland). After dissolving in 25 mL water and diluting with internal standards (Germanium, Scandium and Thallium, all NIST traceable and manufactured and tested under ISO Guide 34 & ISO 17025 guidelines, from Inorganic Ventures, Christiansburg Virginia USA), the sample was analyzed with ICP-MS (Agilent ICP-MS 7700x with ISIS, Agilent Technologies Netherlands B.V. Amstelveen, NL). The quantification was done after calibration with external standards (Selenium, Lead, Cadmium, Nickel, Chromium, Molybdenum, Cobalt, Copper, Iron and Zinc, all from Inorganic Ventures). The duplicate results were averaged and corrected for dry matter content. All results were expressed in mg/kg dm. Results below the detection limit of the ICP-MS (in general, 0.1 mg/kg dm) were reported as 0.05 mg/kg dm.

The toxicologically relevant levels of elements in bovine liver that were used for interpretation of results are listed in Table 2. These values are similar to those generally used by other laboratories (for example [3]).

**Table 2:** Toxicologically relevant levels of elements in calf or cow livers (mg/kg dm).

element	toxic threshold (mg/kg dm)	
	less than 1 year	older than 1 year
copper	> 1000	> 1000
zinc	> 1000	> 500
lead	> 33	> 33
iron	> 2500	> 2000

Statistical analysis: Statistical analysis was done with Stata (StataCorp LLC, Texas 77845–4512, USA). Reference intervals were calculated using the ‘non-parametric method’ described by Geffre *et al* [4]. Median and standard deviation were calculated using the untransformed robust method. Correlations were calculated using Spearman’s rank correlation coefficient. Linear regression was done with year as the independent variable for each element, and data for age-group analysis were corrected to remove year-effects. Regression analysis was done after logarithmic transformation of the element concentration. Residuals were tested with Shapiro Wilk test for normality. Q-Q-plots were used to identify non-normality. The residuals appear similar.

## Results

The median results for each age-group (mean, lower level reference value and upper level reference value with 90% confidence and number of animals) can be seen in Table 3 (for trace elements) and Table 4 (for other elements).

**Table 3:** Results (median and 90% upper and 90% lower level) of trace elements in bovine livers, depending on age of the animal.

Element	(mg/kg dm)	age					
		0–7 days	1–4 weeks	2–6 months	7–12 months	13–24 months	> 2 years
Copper	median	348	324	505	165	115	338
	LL ref value	66	72	13	7	7	7
	UL ref value	715	750	1567	853	713	1066
	number	142	194	220	132	151	701
Cobalt	median	0.1	0.1	0.2	0.2	0.2	0.2
	LL ref value	0.05	0.05	0.1	0.1	0.1	0.1
	UL ref value	0.3	0.8	0.5	0.6	0.5	0.5
	number	142	194	220	132	150	698
Iron	median	594	424	408	478	465	515
	LL ref value	127	124	87	160	157	158
	UL ref value	6686	4086	3085	2070	1400	1945
	number	143	194	220	132	150	699
Molybdenum	median	1.3	1.7	2.2	2.6	2.9	2.8
	LL ref value	0.1	0.8	0.6	0.8	1.2	1.0
	UL ref value	2.3	3.1	3.8	5.3	4.8	4.7
	number	142	194	220	132	151	699
Selenium	median	1.9	1.6	1.6	1.2	1.2	1.6
	LL ref value	0.5	0.7	0.2	0.2	0.2	0.4
	UL ref value	7.1	5.1	4.8	4.4	3.2	4.3
	number	68	130	98	41	60	367
Zinc	median	498	608	267	180	202	185
	LL ref value	114	173	76	77	73	70
	UL ref value	1837	1774	1374	988	970	938
	number	142	194	220	132	150	699

LL ref value Lower limit of reference interval (90%)

UL ref value Upper limit of reference interval (90%)

**Table 4:** Results (median) of other than trace elements in bovine livers, depending on age of the animal.

Element	(mg/kg dm)	age					
		0–7 days	1–4 weeks	2–6 months	7–12 months	13–24 months	> 2 years
Cadmium	median	0.1	0.1	0.1	0.1	0.2	0.2
	LL ref value	0.1	0.1	0.1	0.1	0.1	0.1
	UL ref value	0.1	0.1	0.2	0.5	0.9	0.9
	number	140	194	220	132	150	698
Chromium	median	0.3	0.3	0.3	0.4	0.3	0.3
	LL ref value	0.1	0.1	0.1	0.1	0.1	0.1
	UL ref value	1.1	1.3	1.4	1.1	1.0	1.1
	number	139	194	220	132	150	698
Lead	median	0.2	1.0	1.4	1.8	0.3	0.3
	LL ref value	0.1	0.1	0.1	0.1	0.1	0.1
	UL ref value	1.0	2.0	7.6	22.3	1.1	1.6
	number	140	194	220	132	150	698
Nickel	median	0.2	0.3	0.3	0.3	0.2	0.3
	LL ref value	0.1	0.1	0.1	0.1	0.1	0.1
	UL ref value	0.9	1.6	1.4	0.9	0.5	1.1
	number	140	194	220	132	150	694

LL ref value Lower limit of reference interval (90%)

UL ref value Upper limit of reference interval (90%)

All results are expressed as mg/kg dm.

**Fig 1:**
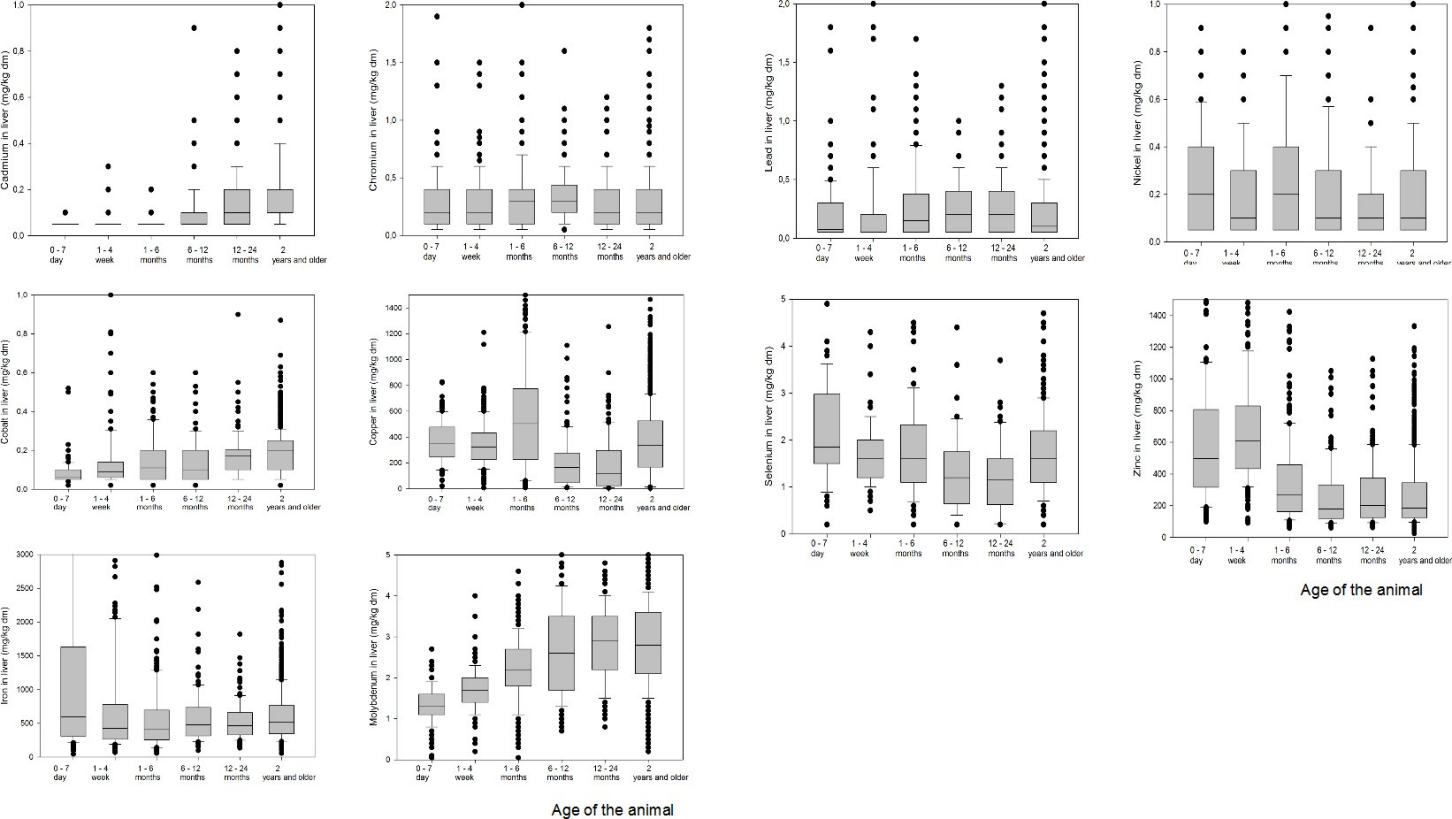
Box plots of elements for each age group. Fig 1A Cadmium, Fig 1B Chromium, Fig 1C Cobalt, Fig 1D Copper, Fig 1E Iron, Fig 1F Lead, Fig 1G Molybdenum, Fig 1H Nickel, Fig 1I Selenium, Fig 1J Zinc.

The results are shown in box-and-whisker-plots for each age group (0–7 days, 1–4 weeks, 1–6 months, 6–12 months, 12–24 months and older than 2 years) in Fig 1.

Cd (Fig 1A) liver concentrations tended to increase with age. Chromium (Fig 1B) remained constant as animals aged. Cobalt (Fig 1C) increased slightly with age. Copper (Fig 1D) was relatively constant in very young animals but decreased after 6 months until the lowest levels were reached at 12–24 months. Animals older than 2 years occasionally had very high copper levels in the liver. Iron (Fig 1E) was high in young animals and decreased rapidly during the first months, remaining stable thereafter. Lead (Fig 1F) tended to remain low with age. Molybdenum (Fig 1G) showed the same pattern as cadmium: low in young animals, higher in older animals. Nickel (Fig 1H) was relatively low in all animals. Selenium followed the same pattern as copper (Fig 1I). The lowest levels were measured in animals of 6 to 24 months old. Zinc (Fig 1J) followed the same pattern as iron: relatively high in young animals compared to older animals.

Correlations between the various elements are shown in Supporting information (S1 Table). There is a significant correlation between trace elements that are added in feed: copper, selenium, cobalt and zinc. Also nickel and chromium are significantly correlated.

**Fig 2:**
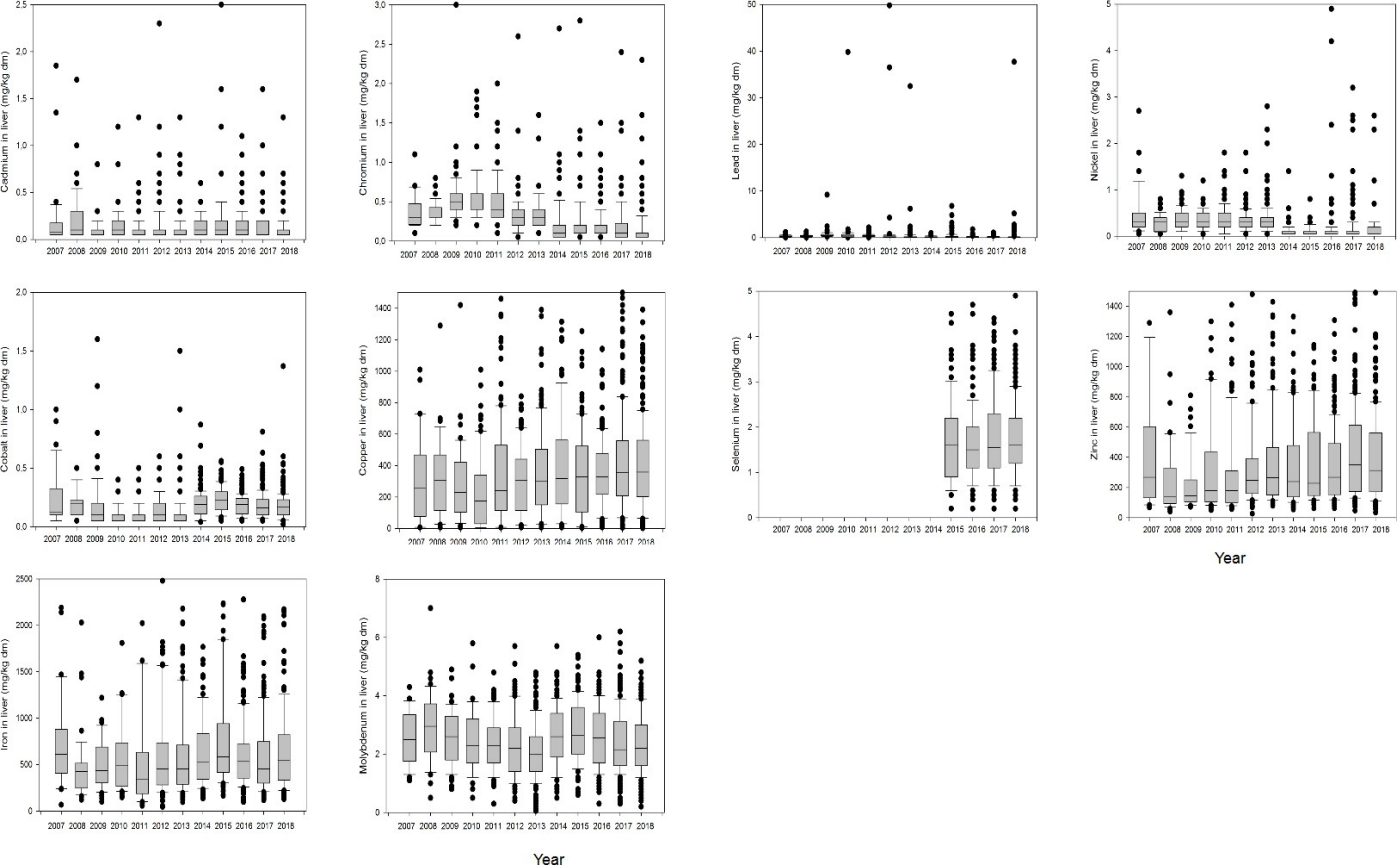
Box plots of elements for each year. Fig 2A Cadmium, Fig 2B Chromium, Fig 2C Cobalt, Fig 2D Copper, Fig 2E Iron, Fig 2F Molybdenum,Fig 2G Lead, Fig 2H Nickel, Fig 2I Selenium, Fig 2J Zinc.

The data are also summarized based on years (2007–2018) (Fig 2).

Chromium (Fig 2B) decreased significantly over the study period. This was also true for nickel (Fig 2I). Copper (Fig 2D) increased significantly over the years. The results of linear regression for the elements with year and age are shown in Table 5. More detailed information can be found in Supporting information (S2 Table).

**Table 5:** Results of linear regression for elements with year.

	element / significance									
	Cd	Cr	Co	Cu	Fe	Pb	Mo	Ni	Se	Zn
P value	0.822	< 0.001	0.134	<0.001	0.833	0.100	0.867	<0.01	0.177	0.001
F (1,1300)	0.05	49.16	2.25	17.56	0.04	2.71	0.03	43.01	1.83	10.45

**Fig 3:**
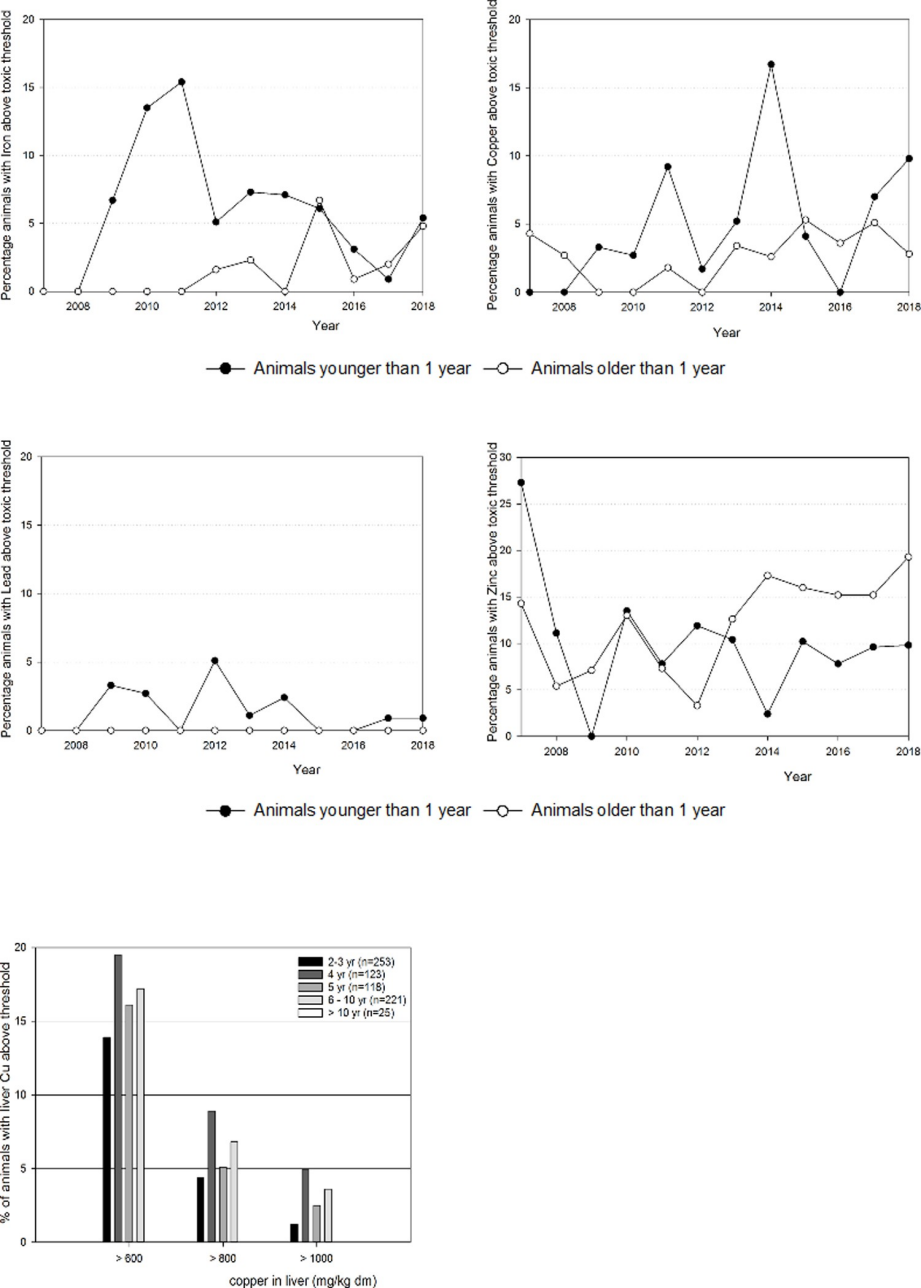
Percentage of animals (young versus older animals) with copper, zinc or iron, and numbers of animals for lead, above the toxic thresholds for young and older animals. Fig 3A Copper, Fig 3B Zinc, Fig 3C Iron, Fig 3D Lead, Fig 3E Animals at different age with copper above a certain threshold limit.

An analysis was made of fraction of livers containing a toxicologically relevant level of one or more elements by comparing two age groups: animals younger than one year and animals older than one year. The elements that were most often at concentrations associated with toxic effects were copper, zinc, iron and lead. The percentage of liver samples with copper, zinc and iron values above the toxicological threshold are shown in Fig 3 while lead is shown as number of cases because the total number was comparatively low.

Toxicologically relevant copper levels in livers (Fig 3A) were more common in animals older than 1 year. The same pattern, although to a lesser extent, was observed for zinc (Fig 3B). Young animals were also more prone to high iron concentrations in the liver (Fig 3C). There was a total of 10 animals with liver lead concentrations above the toxic threshold. All of those animals were aged 12 months or less (Fig 3D). The deaths of 7 animals were attributed to lead poisoning during the study. They were on average 36 days old (SD = 11 days). A further 3 animals had liver lead concentrations above the toxic threshold of 33 mg/kg dm.

The increasing percentage of toxicologically relevant levels of copper was further analyzed. The group aged 2 years and older was split up into five sub-groups: 2–3 years, 4 years, 5 years, 6–10 years, and older than 10 years. The percentage of animals with liver copper concentrations above 600, 800 or 1000 mg/kg dm are shown in Fig 3E. The highest percentages of animals with high liver copper were consistently found in the 4 years group. This was statistically significant in the 1000 mg/kg dm group (P< 0.01).

## Discussion

In this study, we evaluated the concentrations of different trace elements and heavy metals in the livers of cattle based on 6 age groups, and the time period from 2007–2018. Copper and selenium concentrations were correlated. Both elements are relatively low in local forage, and are typically supplemented by farmers and/or feed companies. Therefore, animals living outside on local forage and not supplemented with these trace elements are expected to be relatively low in both copper and selenium [5]. Other positive correlations were found between cadmium and cobalt, cadmium and molybdenum, and cobalt and molybdenum. Young animals tended to have low cadmium, molybdenum and cobalt compared to older animals. Chromium and nickel were also correlated: both are present in stainless steel.

Most elements originate primarily from feed and not from drinking water. About 30% of the cattle in the Netherlands drink surface water. The surface water in the Netherlands is monitored [6]. Elements in surface water are rather low and the contribution of water to the total element uptake is of low significance (less than 10% of total uptake), although this can be completely different in other regions [7, 8]. In general, the concentrations of trace elements in young animals is different from the levels in older animals. Cobalt is incorporated into cobalamin (vitamin B12) by bacteria in the rumen [9]. Vitamin B12 is only really necessary in cattle with a functioning rumen because glucose is metabolized in the liver and the animal relies on vitamin B12 for metabolism of fats and carbohydrates [10, 11]. The concentration typically used to support a diagnosis of cobalt deficiency in older cattle (<0.1 mg / kg ds), is a normal level in a non-ruminating calf. As a consequence, it is important to know the age of tested animals for interpreting liver cobalt concentrations.

The transfer of selenium and copper from the mother to the fetus is well regulated [12–15]. Calves are born with fairly consistent levels of selenium and copper. Graham *et al* [14] found that maternal liver copper concentrations did not vary during gestation. As fetal size increased, fetal liver copper increased, indicating an active transport of copper from the mother to the fetus. Smart and Christensen [15] found that the bovine fetal liver copper concentration was not affected by the dam's age, breed, stage of pregnancy, or copper status. The copper content of grass from the Netherlands is generally low (on average 8.8 mg/kg dm, year 2009–2013, data from Eurofins/BLGG AgroXpertus, The Netherlands) while the target value for optimal health in cattle is 12 to 15 mg/kg dm. Farmers are aware of the low copper concentrations in grass, and are therefore focused on preventing copper deficiencies. Feed companies satisfy this need by adding copper to their concentrates. Farmers occasionally add additional copper, above levels that are optimal, and this can result in a high liver copper concentration after several months or years.

Selenium is mainly present in the selenium-containing enzyme glutathione peroxidase (GSH-Px) in red blood cells [16]. Selenium in the liver remains fairly constant (see Fig 1I) up to the age of 6 months. The pattern of change in liver selenium concentrations (falling from 6 months to 24 months and then increasing again) is comparable to that of copper (shown in Fig 1D), and this pattern was also found in a previous study of GSH-Px [16]. Molybdenum, on the other hand, has a very different pattern. Molybdenum is not considered to be an essential element for cattle, but is present in almost all roughage in the Netherlands. Clover is used increasingly in grass pastures in the Netherlands to reduce the amount of fertilizer required, and molybdenum is an essential element for clover and other plants [17, 18]. Molybdenum absorbed from the feed is stored in the liver. Therefore, the more an animal eats grass, the higher the molybdenum level tends to be in the liver. Molybdenum in feed influences (together with sulphur) the efficiency of absorption of dietary copper. Higher molybdenum and/or sulphur in feed results in lower absorption of copper from the intestinal tract because of the formation of complexes with copper that can’t be absorbed [19].

Young animals have a greater need for iron and zinc compared to older animals [1, 20]. This may be the biological explication for the higher liver concentrations typically found in young animals compared to older animals [21, 22]. The zinc level steadily drops from birth until 2 years because growing animals need more zinc than non-growing animals [23]. A toxicologically relevant level of zinc in an older animal (above 500 mg/kg dm) is considered to be low to normal for young animals [24]. Very high liver zinc concentrations of more than 2000 mg/kg dm were occasionally seen in young animals (less than 75 days of age). These observations of high values remain unexplained.

The iron content of the liver drops immediately after birth due to a high demand for iron to replace fetal hemoglobin with normal hemoglobin [25]. This may explain the decrease in iron levels in the liver after 7 days. The variation of iron in the liver of new-born calves is relatively high, much higher than the variation of copper, zinc or selenium in the liver. Iron supplementation in the form of injections may occasionally be necessary when the mother did not have enough iron. About 30% of the calves in The Netherlands need iron injections (unpublished results, GD Animal Health). Other researchers report even higher estimates, for example that 50% of the new born calves need iron injections [26]. Despite the fact that iron is a relatively harmless element, poisonings still occur because animals may ingest, or are injected with, too much iron.

Nickel is used as a catalyst for hydrogenation of fat [27] (for example sunflower seed oil and soybean oil) and nickel may therefore occur in oil products as a contaminant. Especially young animals get additional fat, in the form of vegetable oil products, in their feed. However, poisoning due to nickel were not found. Poisoning by chromium was also not found. Nickel and chromium are of concern for public health [28] but can also influence the metabolism of other elements [29]. The uptake of these metals from the environment appears to have been reduced significantly compared to previous years and studies [30–34]. Because nickel and chromium are both found in stainless steel, a positive correlation between nickel and chromium was often described. There was a tendency for both nickel and chromium to decrease in the last years, indicating that contamination of feed with particles of stainless steel is decreasing.

In the past, farmers used lead-containing paint on doors and walls. This is no longer allowed in the Netherlands. Lead is palatable to calves [35] and this may be a factor in the occurrence of poisoning in young animals. Copper, iron and zinc toxicosis were also diagnosed. There was an increasing trend in poisoning from these metals during the study period. This trend is in agreement with other studies and reports [3, 30, 36–39].

In conclusion: the measurement of trace elements and metals in livers of cattle is not only important for the individual animal and owner, but also provides important monitoring information for producers of concentrates, governments and farmer organizations. Due to consistent patterns of variations in liver element concentrations associated with age, the age of animals should be known for proper interpretation of laboratory results. The revelation of poisoning trends, particularly increasing incidences of poisoning by copper, iron and zinc, could be of value to feed producers and mineral suppliers to adjust trace element levels and reduce uptake of these elements where necessary.

## Supporting information

S1 Table Correlation between various elements. (DOCX)

S2 Table Linear regression results of elements with year and age. (DOCX)
