# Supplementary material for: Levels of trace elements and potential toxic elements in bovine livers: A trend analysis from 2007 to 2018
Source: PLoS One. 2019 Apr 9;14(4):e0214584. doi: 10.1371/journal.pone.0214584 (PMC6456170; doi:10.1371/journal.pone.0214584)
Supplement: S1 Table — (DOCX) [file pone.0214584.s001.docx]

**S1 Table Correlation matrix of elements from liver (all years and ages combined) (Spearman’s rank correlation)**

|  | Cd | Cr | Co | Cu | Fe | Pb | Mo | Ni | Se |
| --- | --- | --- | --- | --- | --- | --- | --- | --- | --- |
| Cr | 0.0748 |  |  |  |  |  |  |  |  |
|  | 0.0395 |  |  |  |  |  |  |  |  |
| Co | 0.5234 | 0.0606 |  |  |  |  |  |  |  |
|  | 0.0000 | 0.0955 |  |  |  |  |  |  |  |
| Cu | -0.0529 | 0.0243 | 0.2644 |  |  |  |  |  |  |
|  | 0.1453 | 0.5034 | 0.0000 |  |  |  |  |  |  |
| Fe | 0.1649 | 0.0704 | 0.0674 | -0.0073 |  |  |  |  |  |
|  | 0.0000 | 0.0526 | 0.0638 | 0.8410 |  |  |  |  |  |
| Pb | 0.3523 | 0.2229 | 0.1365 | -0.3253 | 0.2637 |  |  |  |  |
|  | 0.0000 | 0.0000 | 0.0002 | 0.0000 | 0.0000 |  |  |  |  |
| Mo | 0.5316 | 0.0307 | 0.6366 | 0.0958 | -0.0414 | 0.1875 |  |  |  |
|  | 0.0000 | 0.3981 | 0.0000 | 0.0083 | 0.2549 | 0.0000 |  |  |  |
| Ni | -0.0296 | 0.2692 | 0.0439 | 0.0747 | 0.0237 | 0.0245 | 0.0038 |  |  |
|  | 0.4151 | 0.0000 | 0.2274 | 0.0397 | 0.5145 | 0.4999 | 0.9176 |  |  |
| Se | -0.0811 | 0.0318 | 0.1873 | 0.6185 | -0.0194 | -0.3017 | 0.0139 | 0.1112 |  |
|  | 0.0255 | 0.3823 | 0.0000 | 0.0000 | 0.5932 | 0.0000 | 0.7016 | 0.0022 |  |
| Zn | -0.2313 | 0.1040 | -0.2600 | 0.0974 | 0.2394 | 0.0054 | -0.2782 | 0.0574 | 0.0399 |
|  | 0.0000 | 0.0041 | 0.0000 | 0.0073 | 0.0000 | 0.8819 | 0.0000 | 0.1141 | 0.2725 |
