# Supplementary material for: Levels of trace elements and potential toxic elements in bovine livers: A trend analysis from 2007 to 2018
Source: PLoS One. 2019 Apr 9;14(4):e0214584. doi: 10.1371/journal.pone.0214584 (PMC6456170; doi:10.1371/journal.pone.0214584)
Supplement: S2 Table — (DOCX) [file pone.0214584.s002.docx]

**S2 Regression analysis**

**year (2007 – 2018) with element concentration after log transformation in liver of cow**

**Cd**

Source | SS df MS Number of obs = 1,534

-------------+---------------------------------- F(1, 1532) = 3.98

Model | 37.9488116 1 37.9488116 Prob > F = 0.0463

Residual | 14622.4997 1,532 9.54471259 R-squared = 0.0026

-------------+---------------------------------- Adj R-squared = 0.0019

Total | 14660.4485 1,533 9.56324103 Root MSE = 3.0895

------------------------------------------------------------------------------

year | Coef. Std. Err. t P>|t| [95% Conf. Interval]

-------------+----------------------------------------------------------------

LCd | -.2028588 .1017364 -1.99 0.046 -.4024161 -.0033015

_cons | 2013.656 .2567925 7841.57 0.000 2013.152 2014.16

------------------------------------------------------------------------------

Shapiro-Wilk W test for normal data

Variable | Obs W V z Prob>z

-------------+------------------------------------------------------

res | 1,534 0.94071 55.232 10.104 0.00000

**Cr**

Source | SS df MS Number of obs = 1,533

-------------+---------------------------------- F(1, 1531) = 696.02

Model | 4580.79677 1 4580.79677 Prob > F = 0.0000

Residual | 10076.2026 1,531 6.58145172 R-squared = 0.3125

-------------+---------------------------------- Adj R-squared = 0.3121

Total | 14656.9993 1,532 9.56723195 Root MSE = 2.5654

------------------------------------------------------------------------------

year | Coef. Std. Err. t P>|t| [95% Conf. Interval]

-------------+----------------------------------------------------------------

LCr | -1.868242 .0708147 -26.38 0.000 -2.007146 -1.729338

_cons | 2011.134 .1315155 1.5e+04 0.000 2010.876 2011.392

------------------------------------------------------------------------------

Shapiro-Wilk W test for normal data

Variable | Obs W V z Prob>z

-------------+------------------------------------------------------

res | 1,533 0.99406 5.533 4.309 0.00001

**Co**

Source | SS df MS Number of obs = 1,536

-------------+---------------------------------- F(1, 1534) = 50.63

Model | 472.614918 1 472.614918 Prob > F = 0.0000

Residual | 14319.3851 1,534 9.33467085 R-squared = 0.0320

-------------+---------------------------------- Adj R-squared = 0.0313

Total | 14792 1,535 9.63648208 Root MSE = 3.0553

------------------------------------------------------------------------------

year | Coef. Std. Err. t P>|t| [95% Conf. Interval]

-------------+----------------------------------------------------------------

LCo | .7694077 .1081315 7.12 0.000 .5573064 .9815089

_cons | 2015.684 .2325323 8668.40 0.000 2015.228 2016.14

------------------------------------------------------------------------------

Shapiro-Wilk W test for normal data

Variable | Obs W V z Prob>z

-------------+------------------------------------------------------

res | 1,536 0.96463 32.982 8.806 0.00000

**Cu**

Source | SS df MS Number of obs = 1,540

-------------+---------------------------------- F(1, 1538) = 39.61

Model | 371.667568 1 371.667568 Prob > F = 0.0000

Residual | 14432.1318 1,538 9.38370077 R-squared = 0.0251

-------------+---------------------------------- Adj R-squared = 0.0245

Total | 14803.7994 1,539 9.61910289 Root MSE = 3.0633

------------------------------------------------------------------------------

year | Coef. Std. Err. t P>|t| [95% Conf. Interval]

-------------+----------------------------------------------------------------

LCu | .3800867 .0603938 6.29 0.000 .2616238 .4985496

_cons | 2012.08 .3346783 6011.98 0.000 2011.423 2012.736

------------------------------------------------------------------------------

Shapiro-Wilk W test for normal data

Variable | Obs W V z Prob>z

-------------+------------------------------------------------------

res | 1,540 0.95361 43.368 9.496 0.00000

**Fe**

Source | SS df MS Number of obs = 1,538

-------------+---------------------------------- F(1, 1536) = 9.03

Model | 85.9662826 1 85.9662826 Prob > F = 0.0027

Residual | 14624.6325 1,536 9.52124515 R-squared = 0.0058

-------------+---------------------------------- Adj R-squared = 0.0052

Total | 14710.5988 1,537 9.57098167 Root MSE = 3.0857

------------------------------------------------------------------------------

year | Coef. Std. Err. t P>|t| [95% Conf. Interval]

-------------+----------------------------------------------------------------

LFe | .3132994 .1042659 3.00 0.003 .1087808 .5178179

_cons | 2012.183 .6548157 3072.90 0.000 2010.898 2013.467

------------------------------------------------------------------------------

Shapiro-Wilk W test for normal data

Variable | Obs W V z Prob>z

-------------+------------------------------------------------------

res | 1,538 0.94227 53.905 10.043 0.00000

**Pb**

Source | SS df MS Number of obs = 1,534

-------------+---------------------------------- F(1, 1532) = 437.11

Model | 3254.39868 1 3254.39868 Prob > F = 0.0000

Residual | 11406.0498 1,532 7.44520223 R-squared = 0.2220

-------------+---------------------------------- Adj R-squared = 0.2215

Total | 14660.4485 1,533 9.56324103 Root MSE = 2.7286

------------------------------------------------------------------------------

year | Coef. Std. Err. t P>|t| [95% Conf. Interval]

-------------+----------------------------------------------------------------

LPb | -1.293715 .0618788 -20.91 0.000 -1.415091 -1.172339

_cons | 2011.655 .1379304 1.5e+04 0.000 2011.384 2011.925

------------------------------------------------------------------------------

Shapiro-Wilk W test for normal data

Variable | Obs W V z Prob>z

-------------+------------------------------------------------------

res | 1,534 0.97643 21.951 7.780 0.00000

**Mo**

Source | SS df MS Number of obs = 1,538

-------------+---------------------------------- F(1, 1536) = 0.17

Model | 1.62180291 1 1.62180291 Prob > F = 0.6812

Residual | 14750.0537 1,536 9.60289958 R-squared = 0.0001

-------------+---------------------------------- Adj R-squared = -0.0005

Total | 14751.6756 1,537 9.59770693 Root MSE = 3.0989

------------------------------------------------------------------------------

year | Coef. Std. Err. t P>|t| [95% Conf. Interval]

-------------+----------------------------------------------------------------

LMo | -.0614893 .1496241 -0.41 0.681 -.3549784 .2319998

_cons | 2014.181 .1413008 1.4e+04 0.000 2013.904 2014.459

------------------------------------------------------------------------------

Shapiro-Wilk W test for normal data

Variable | Obs W V z Prob>z

-------------+------------------------------------------------------

res | 1,538 0.92960 65.732 10.543 0.00000

**Ni**

Source | SS df MS Number of obs = 1,530

-------------+---------------------------------- F(1, 1528) = 486.19

Model | 3532.66172 1 3532.66172 Prob > F = 0.0000

Residual | 11102.5148 1,528 7.26604368 R-squared = 0.2414

-------------+---------------------------------- Adj R-squared = 0.2409

Total | 14635.1765 1,529 9.57173085 Root MSE = 2.6956

------------------------------------------------------------------------------

year | Coef. Std. Err. t P>|t| [95% Conf. Interval]

-------------+----------------------------------------------------------------

LNi | -1.498932 .0679798 -22.05 0.000 -1.632276 -1.365589

_cons | 2011.174 .1510239 1.3e+04 0.000 2010.878 2011.47

------------------------------------------------------------------------------

Shapiro-Wilk W test for normal data

Variable | Obs W V z Prob>z

-------------+------------------------------------------------------

res | 1,530 0.98999 9.305 5.617 0.00000

**Se**

Source | SS df MS Number of obs = 764

-------------+---------------------------------- F(1, 762) = 1.83

Model | 2.08428562 1 2.08428562 Prob > F = 0.1766

Residual | 868.275662 762 1.13946937 R-squared = 0.0024

-------------+---------------------------------- Adj R-squared = 0.0011

Total | 870.359948 763 1.14070766 Root MSE = 1.0675

------------------------------------------------------------------------------

year | Coef. Std. Err. t P>|t| [95% Conf. Interval]

-------------+----------------------------------------------------------------

LSe | .0840322 .0621324 1.35 0.177 -.0379389 .2060033

_cons | 2016.767 .0454152 4.4e+04 0.000 2016.678 2016.857

------------------------------------------------------------------------------

Shapiro-Wilk W test for normal data

Variable | Obs W V z Prob>z

-------------+------------------------------------------------------

res | 764 0.88064 58.928 9.982 0.00000

**Zn**

Source | SS df MS Number of obs = 1,537

-------------+---------------------------------- F(1, 1535) = 53.53

Model | 495.405511 1 495.405511 Prob > F = 0.0000

Residual | 14205.3531 1,535 9.2543017 R-squared = 0.0337

-------------+---------------------------------- Adj R-squared = 0.0331

Total | 14700.7586 1,536 9.57080639 Root MSE = 3.0421

------------------------------------------------------------------------------

year | Coef. Std. Err. t P>|t| [95% Conf. Interval]

-------------+----------------------------------------------------------------

LZn | .7243809 .0990053 7.32 0.000 .530181 .9185808

_cons | 2010.082 .5597335 3591.14 0.000 2008.984 2011.18

------------------------------------------------------------------------------

Shapiro-Wilk W test for normal data

Variable | Obs W V z Prob>z

-------------+------------------------------------------------------

res | 1,537 0.95787 39.310 9.248 0.00000

**linear regression of age category (1 – 7 days, 1 – 4 weeks, 2 – 6 months, 7 – 12 months, 13 – 24 months, > 2 years) with element concentration in liver of cow after log transformation**

**Cd**

Source | SS df MS Number of obs = 1,534

-------------+---------------------------------- F(1, 1532) = 662.70

Model | 1516.74184 1 1516.74184 Prob > F = 0.0000

Residual | 3506.34029 1,532 2.28873387 R-squared = 0.3020

-------------+---------------------------------- Adj R-squared = 0.3015

Total | 5023.08214 1,533 3.27663545 Root MSE = 1.5129

------------------------------------------------------------------------------

agecat | Coef. Std. Err. t P>|t| [95% Conf. Interval]

-------------+----------------------------------------------------------------

LCd | 1.28248 .0498187 25.74 0.000 1.18476 1.3802

_cons | 7.41828 .1257473 58.99 0.000 7.171625 7.664935

------------------------------------------------------------------------------

Shapiro-Wilk W test for normal data

Variable | Obs W V z Prob>z

-------------+------------------------------------------------------

res | 1,534 0.98718 11.944 6.247 0.00000

**Cr**

Source | SS df MS Number of obs = 1,533

-------------+---------------------------------- F(1, 1531) = 2.89

Model | 9.4506563 1 9.4506563 Prob > F = 0.0892

Residual | 5002.48411 1,531 3.26746186 R-squared = 0.0019

-------------+---------------------------------- Adj R-squared = 0.0012

Total | 5011.93477 1,532 3.27149789 Root MSE = 1.8076

------------------------------------------------------------------------------

agecat | Coef. Std. Err. t P>|t| [95% Conf. Interval]

-------------+----------------------------------------------------------------

LCr | -.0848582 .0498962 -1.70 0.089 -.1827304 .0130141

_cons | 4.203211 .0926662 45.36 0.000 4.021445 4.384977

------------------------------------------------------------------------------

Shapiro-Wilk W test for normal data

Variable | Obs W V z Prob>z

-------------+------------------------------------------------------

res | 1,533 0.83411 154.433 12.693 0.00000

**Co**

Source | SS df MS Number of obs = 1,536

-------------+---------------------------------- F(1, 1534) = 228.57

Model | 654.272509 1 654.272509 Prob > F = 0.0000

Residual | 4391.06082 1,534 2.86249076 R-squared = 0.1297

-------------+---------------------------------- Adj R-squared = 0.1291

Total | 5045.33333 1,535 3.28686211 Root MSE = 1.6919

------------------------------------------------------------------------------

agecat | Coef. Std. Err. t P>|t| [95% Conf. Interval]

-------------+----------------------------------------------------------------

LCo | .9052784 .059879 15.12 0.000 .7878249 1.022732

_cons | 6.167435 .1287674 47.90 0.000 5.914856 6.420013

------------------------------------------------------------------------------

Shapiro-Wilk W test for normal data

Variable | Obs W V z Prob>z

-------------+------------------------------------------------------

res | 1,536 0.94348 52.711 9.987 0.00000

**Cu**

Source | SS df MS Number of obs = 1,540

-------------+---------------------------------- F(1, 1538) = 31.86

Model | 102.56194 1 102.56194 Prob > F = 0.0000

Residual | 4951.52832 1,538 3.21945925 R-squared = 0.0203

-------------+---------------------------------- Adj R-squared = 0.0197

Total | 5054.09026 1,539 3.28400927 Root MSE = 1.7943

------------------------------------------------------------------------------

agecat | Coef. Std. Err. t P>|t| [95% Conf. Interval]

-------------+----------------------------------------------------------------

LCu | -.1996634 .035375 -5.64 0.000 -.2690518 -.130275

_cons | 5.412951 .1960343 27.61 0.000 5.028429 5.797474

------------------------------------------------------------------------------

Shapiro-Wilk W test for normal data

Variable | Obs W V z Prob>z

-------------+------------------------------------------------------

res | 1,540 0.86907 122.394 12.110 0.00000

**Fe**

Source | SS df MS Number of obs = 1,538

-------------+---------------------------------- F(1, 1536) = 1.72

Model | 5.66107282 1 5.66107282 Prob > F = 0.1898

Residual | 5053.55934 1,536 3.2900777 R-squared = 0.0011

-------------+---------------------------------- Adj R-squared = 0.0005

Total | 5059.22042 1,537 3.29162031 Root MSE = 1.8139

------------------------------------------------------------------------------

agecat | Coef. Std. Err. t P>|t| [95% Conf. Interval]

-------------+----------------------------------------------------------------

LFe | -.0803979 .0612913 -1.31 0.190 -.2006213 .0398255

_cons | 4.83351 .3849243 12.56 0.000 4.078478 5.588543

------------------------------------------------------------------------------

Shapiro-Wilk W test for normal data

Variable | Obs W V z Prob>z

-------------+------------------------------------------------------

res | 1,538 0.82392 164.403 12.852 0.00000

**Pb**

Source | SS df MS Number of obs = 1,534

-------------+---------------------------------- F(1, 1532) = 3.71

Model | 12.1403436 1 12.1403436 Prob > F = 0.0542

Residual | 5010.94179 1,532 3.27084974 R-squared = 0.0024

-------------+---------------------------------- Adj R-squared = 0.0018

Total | 5023.08214 1,533 3.27663545 Root MSE = 1.8085

------------------------------------------------------------------------------

agecat | Coef. Std. Err. t P>|t| [95% Conf. Interval]

-------------+----------------------------------------------------------------

LPb | .0790167 .0410141 1.93 0.054 -.0014331 .1594665

_cons | 4.489693 .0914223 49.11 0.000 4.310367 4.669019

------------------------------------------------------------------------------

Shapiro-Wilk W test for normal data

Variable | Obs W V z Prob>z

-------------+------------------------------------------------------

res | 1,534 0.87453 116.876 11.992 0.00000

**Mo**

Source | SS df MS Number of obs = 1,538

-------------+---------------------------------- F(1, 1536) = 380.21

Model | 1001.72053 1 1001.72053 Prob > F = 0.0000

Residual | 4046.83149 1,536 2.63465592 R-squared = 0.1984

-------------+---------------------------------- Adj R-squared = 0.1979

Total | 5048.55202 1,537 3.28467926 Root MSE = 1.6232

------------------------------------------------------------------------------

agecat | Coef. Std. Err. t P>|t| [95% Conf. Interval]

-------------+----------------------------------------------------------------

LMo | 1.528177 .0783722 19.50 0.000 1.374449 1.681905

_cons | 3.138431 .0740126 42.40 0.000 2.993255 3.283608

------------------------------------------------------------------------------

Shapiro-Wilk W test for normal data

Variable | Obs W V z Prob>z

-------------+------------------------------------------------------

res | 1,538 0.95803 39.188 9.240 0.00000

**Ni**

Source | SS df MS Number of obs = 1,530

-------------+---------------------------------- F(1, 1528) = 8.14

Model | 26.568005 1 26.568005 Prob > F = 0.0044

Residual | 4985.43199 1,528 3.26271727 R-squared = 0.0053

-------------+---------------------------------- Adj R-squared = 0.0046

Total | 5012 1,529 3.27795945 Root MSE = 1.8063

------------------------------------------------------------------------------

agecat | Coef. Std. Err. t P>|t| [95% Conf. Interval]

-------------+----------------------------------------------------------------

LNi | -.1299901 .0455534 -2.85 0.004 -.2193439 -.0406364

_cons | 4.076365 .1012014 40.28 0.000 3.877857 4.274873

------------------------------------------------------------------------------

Shapiro-Wilk W test for normal data

Variable | Obs W V z Prob>z

-------------+------------------------------------------------------

res | 1,530 0.85417 135.517 12.363 0.00000

**Se**

Source | SS df MS Number of obs = 764

-------------+---------------------------------- F(1, 762) = 9.75

Model | 33.9478337 1 33.9478337 Prob > F = 0.0019

Residual | 2653.6019 762 3.4824172 R-squared = 0.0126

-------------+---------------------------------- Adj R-squared = 0.0113

Total | 2687.54974 763 3.52234566 Root MSE = 1.8661

------------------------------------------------------------------------------

agecat | Coef. Std. Err. t P>|t| [95% Conf. Interval]

-------------+----------------------------------------------------------------

LSe | -.3391354 .1086194 -3.12 0.002 -.5523643 -.1259066

_cons | 4.434103 .0793945 55.85 0.000 4.278246 4.589961

------------------------------------------------------------------------------

Shapiro-Wilk W test for normal data

Variable | Obs W V z Prob>z

-------------+------------------------------------------------------

res | 764 0.84402 77.010 10.638 0.00000

**Zn**

Source | SS df MS Number of obs = 1,537

-------------+---------------------------------- F(1, 1535) = 350.72

Model | 938.876123 1 938.876123 Prob > F = 0.0000

Residual | 4109.23318 1,535 2.67702487 R-squared = 0.1860

-------------+---------------------------------- Adj R-squared = 0.1855

Total | 5048.1093 1,536 3.28652949 Root MSE = 1.6362

------------------------------------------------------------------------------

agecat | Coef. Std. Err. t P>|t| [95% Conf. Interval]

-------------+----------------------------------------------------------------

LZn | -.9972194 .0532492 -18.73 0.000 -1.101668 -.8927706

_cons | 9.917832 .3010479 32.94 0.000 9.327323 10.50834

------------------------------------------------------------------------------

Shapiro-Wilk W test for normal data

Variable | Obs W V z Prob>z

-------------+------------------------------------------------------

res | 1,537 0.97035 27.667 8.363 0.00000
